# Structure–Activity Relationship Studies Using Natural and Synthetic Okadaic Acid/Dinophysistoxin Toxins

**DOI:** 10.3390/md14110207

**Published:** 2016-11-04

**Authors:** Michael J. Twiner, Gregory J. Doucette, Yucheng Pang, Chao Fang, Craig J. Forsyth, Christopher O. Miles

**Affiliations:** 1 School of Medicine, Wayne State University, Detroit, MI 48201, USA; 2 Department of Natural Sciences, University of Michigan, Dearborn, MI 48128, USA; 3 Marine Biotoxins Program, Center for Coastal Environmental Health and Biomolecular Research, NOAA/National Ocean Service, Charleston, SC 29412, USA; greg.doucette@noaa.gov; 4 Department of Chemistry, The Ohio State University, Columbus, OH 43220, USA; yucheng.pang@bioduro.com (Y.P.); chaofangphd@yahoo.com (C.F.); forsyth@chemistry.ohio-state.edu (C.J.F.); 5 Section for Chemistry and Toxicology, Norwegian Veterinary Institute, Oslo 0454, Norway; chris.miles@vetinst.no

**Keywords:** cytotoxicity, diarrheic shellfish poisoning (DSP), dinophysistoxin (DTX), harmful algal bloom (HAB), okadaic acid (OA), protein phosphatase (PP), structure–activity relationship (SAR)

## Abstract

Okadaic acid (OA) and the closely related dinophysistoxins (DTXs) are algal toxins that accumulate in shellfish and are known serine/threonine protein phosphatase (ser/thr PP) inhibitors. Phosphatases are important modulators of enzyme activity and cell signaling pathways. However, the interactions between the OA/DTX toxins and phosphatases are not fully understood. This study sought to identify phosphatase targets and characterize their structure–activity relationships (SAR) with these algal toxins using a combination of phosphatase activity and cytotoxicity assays. Preliminary screening of 21 human and yeast phosphatases indicated that only three ser/thr PPs (PP2a, PP1, PP5) were inhibited by physiologically saturating concentrations of DTX2 (200 nM). SAR studies employed naturally-isolated OA, DTX1, and DTX2, which vary in degree and/or position of methylation, in addition to synthetic 2-*epi*-DTX2. OA/DTX analogs induced cytotoxicity and inhibited PP activity with a relatively conserved order of potency: OA = DTX1 ≥ DTX2 >> 2-*epi*-DTX. The PPs were also differentially inhibited with sensitivities of PP2a > PP5 > PP1. These findings demonstrate that small variations in OA/DTX toxin structures, particularly at the head region (i.e., C1/C2), result in significant changes in toxicological potency, whereas changes in methylation at C31 and C35 (tail region) only mildly affect potency. In addition to this being the first study to extensively test OA/DTX analogs’ activities towards PP5, these data will be helpful for accurately determining toxic equivalence factors (TEFs), facilitating molecular modeling efforts, and developing highly selective phosphatase inhibitors.

## 1. Introduction

Members of the okadaic acid (OA) and dinophysistoxin (DTX) class of marine algal toxins represent a health risk due to contamination of edible seafood [1]. Diarrhetic shellfish poisoning (DSP) is a syndrome in humans caused by the ingestion of shellfish contaminated by OA and DTX produced by dinoflagellates in the genera *Dinophysis* and *Prorocentrum* [2,3]. The OA/DTX toxins are distributed globally, with thousands of people exposed annually. For the first time, people fell ill during 2011 in the Pacific northwest region of the United States due to consumption of shellfish containing high levels of DTX1 [4,5]. Symptoms of DSP intoxication include rapid onset of diarrhea, nausea, vomiting, and abdominal pain, with resolution within a few days [6]. OA has been associated with mutagenic, carcinogenic, and immunosuppressive effects [7]. 

OA/DTXs are known to reversibly inhibit serine/threonine protein phosphatases (ser/thr PPs; i.e., PP2a, PP1) [8]. Protein phosphorylation is regulated by tightly controlled kinases and phosphatases and plays an essential role in many eukaryotic cellular processes. Phosphatases fall into two classes based on substrate selectivity; the PPs and the protein tyrosine phosphatases (PTPs). PPs control a plethora of cellular signaling pathways including cell growth [9] and death [10]. Based on the relatively conserved sequence homology among PPs [11], particularly with respect to the catalytic region, other PPs including PP5 [12,13] appear to be targeted by OA/DTXs in a similar manner [14].

**Figure 1:**
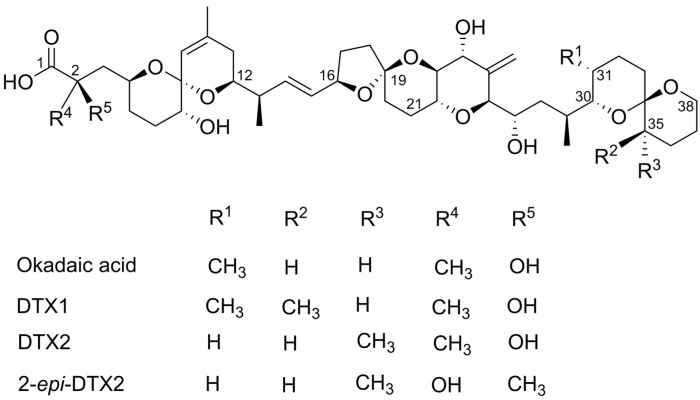
Chemical structures of the okadaic acid/ dinophysistoxin (OA/DTX) class.

The elegant crystal structures of OA bound by PP2a [11] and PP1 [15] have identified two important molecular recognition sites. There is a bimetallic groove that accommodates the carboxylate-bearing C3–C12 spiroketal head region of OA/DTX and a distal hydrophobic groove that hosts the terminal C30–C38 tail spiroketal [15,16]. The carboxylic acid head region of OA that binds close to the active site [11,17] appears to be the primary pharmacophore, as structural modifications at C1 and C2 can reduce inhibitory potencies by several orders of magnitude [14,18]. On the other hand, structural and stereochemical modifications within the tail region of OA/DTX have weaker yet still significant effect on binding to the hydrophobic groove with PP2a [17]. Similarly, OA, DTX1, and DTX2 (Figure 1) each show widely varying effects in mammals despite very small variations in their molecular structure [19].

The OA toxin class has become an important tool used to examine the role of PPs in cellular function and disease. Studying the mechanistic effects of OA and DTXs on their targets is important for understanding the pharmacological properties of these compounds, making them useful for investigating cell signaling pathways and, under some circumstances, PP inhibitors may represent targets for drug discovery [20]. Published data provide some activity-based and atomic-level resolution of essential PP binding sites, but these are yet to be fully characterized. Furthermore, in recent years new phosphatases have been identified. As such, our understanding of the effects of OA/DTX analogs towards many of these phosphatases is incomplete. These critical gaps in our knowledge justify the reassessment and expanded study of the inhibitory effects of OA/DTXs towards phosphatases, as well as the rational design of new synthetic OA/DTX analogs for SAR studies to exploit differential binding site topologies. Through the use of phosphatase activity assays and cytotoxicity assays, we sought to more completely characterize the inhibitory interactions of the OA/DTXs towards PPs that may help regulatory agencies better assess human health risks associated with DSP toxin-contaminated seafood. 

## 2. Results

### 2.1. Cytotoxicty

**Figure 2:**
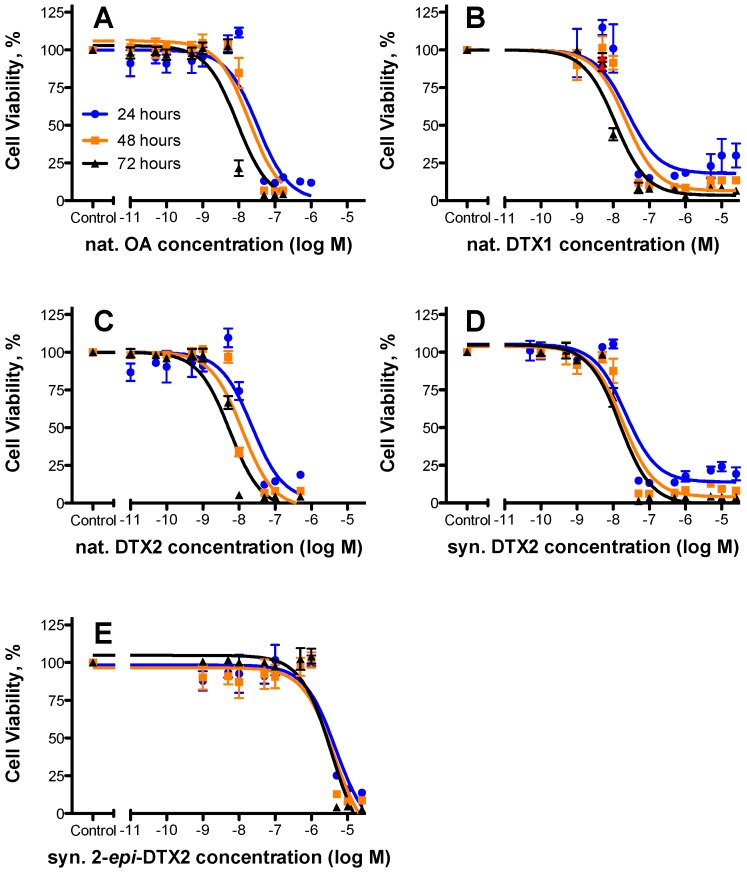
Effect of various diarrheic shellfish poisoning (DSP) toxins on T lymphocyte cell viability. Jurkat T cells were exposed to various concentrations of (**A**) natural OA; (**B**) natural DTX1; (**C**) natural DTX2; (**D**) synthetic DTX2; and (**E**) synthetic 2-*epi*-DTX2 for 24, 48, or 72 h and viability was assessed using the MTS assay. All data (mean ± SE; *n* = 2 or 3) were normalized to the control. Three parameters, variable slope, non-linear dose-response analysis was performed and EC_50_ and 95% confidence interval values were calculated (Table 1).

Natural OA, DTX2, and DTX1 were tested in parallel with synthetic DTX2 and 2-*epi*-DTX2 for cytotoxicity using T lymphocyte cells. All toxins were shown to reduce the viability of these cells in a concentration- and time-dependent manner (Figure 2). Although EC_50_ values were dynamic and decreased with time of exposure, the order of potency remained relatively constant: nat. OA ≈ nat. DTX2 ≈ nat. DTX1 ≈ syn. DTX2 >> syn. 2-*epi*-DTX (Table 1) with 48 h EC_50_ values of 19.5, 12.3, 22.2, 18.2, and 4250 nM, respectively. Although the EC_50_ values for nat. DTX2 appear to be lower than many of the other treatments, overlapping 95% confidence intervals for the nat. OA, syn. DTX2, and nat. DTX1 treatments suggest that they are not statistically different. However, the potency of nat. DTX2 was significantly greater than syn. DTX2 at 72 h.

Using the 48 h cytotoxicity data, relative potencies or toxic equivalence factors (TEFs) (relative to OA) are 1.6 (nat. DTX2), 1.1 (syn. DTX2), 0.9 (DTX1), and 0.004 (2-*epi*-DTX2). These studies demonstrate that 2-*epi*-DTX2 is more than 2 orders of magnitude less potent than the corresponding DTX2.

### 2.2. Protein Phosphatase Inhibition

Preliminary studies conducted on 21 different protein phosphatases, dual specificity phosphatases and tyrosine phosphatases (CD45, DUSP22, HePTP, LMPTP-A, LMPTP-B, MKP5, PP1α, PP2a, PP5, PTPMEG1, PTP-MEG2, PTP-1B, PTPN22, PTPβ, RPTPμ, SHP-1, SHP-2, TCPTP, TMDP, VHR, and YopH) (PhosphataseProfiler, EMD Millipore Corp, Darmstadt, Germany) in the presence 200 nM DTX2 (*n* = 2) indicated that only PP1α, PP2a, and PP5 were inhibited (Figure S1). Based on % inhibition to this single concentration of DTX2, the order of sensitivity in this preliminary study was PP2a > PP5 > PP1.

**Figure 3:**
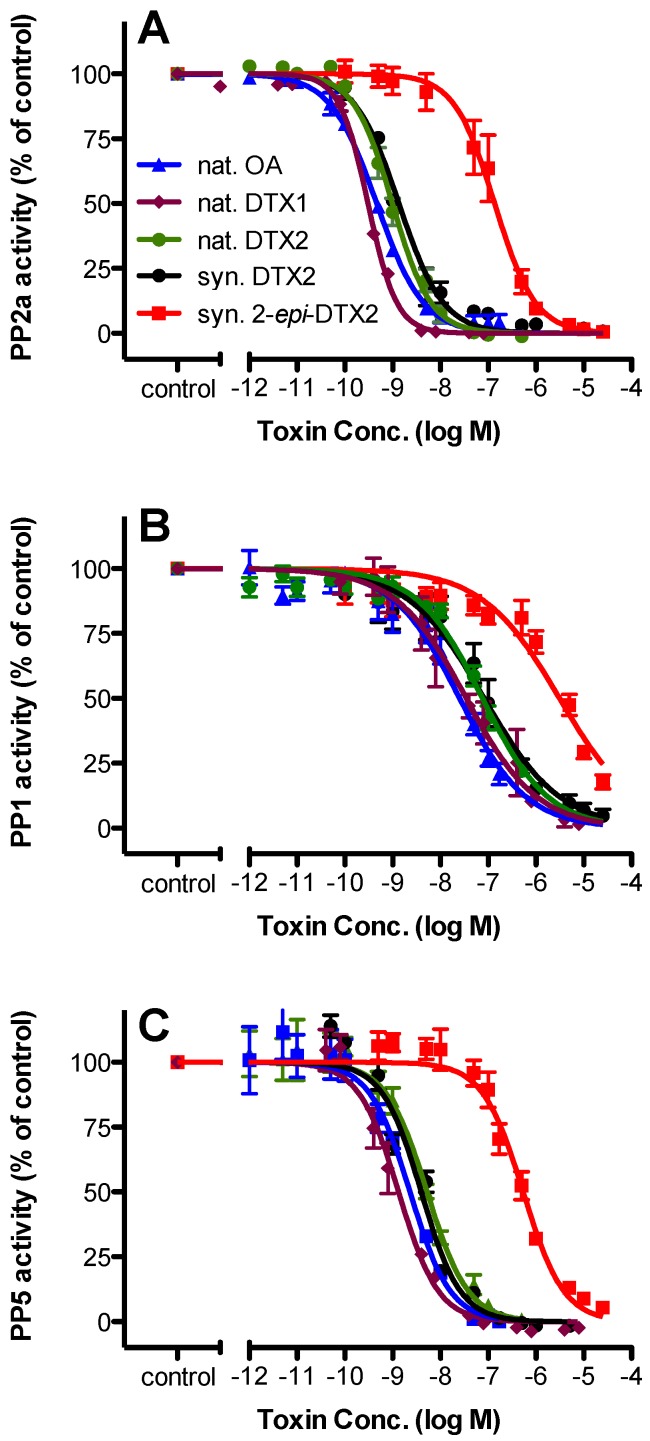
Effect of various DSP toxins on protein phosphatase activity. The activities of protein phosphatases (**A**) PP2a, (**B**) PP1, and (**C**) PP5 were assessed in the presence of various concentrations of natural OA, natural DTX1, natural DTX2, synthetic DTX2, or synthetic 2-*epi*-DTX2. All data (mean ± SE; *n* = 3 or 4) were normalized to the control. Three-parameter, variable slope, non-linear dose-response analysis was performed and IC_50_ and 95% confidence interval values were calculated (Table 2). Note: Some of these data have been published previously in Pang et al. [21].

Natural OA, DTX1, and DTX2 were more thoroughly characterized in parallel with synthesized DTX2 and 2-*epi*-DTX2 for PP2a, PP1, and PP5 inhibition potential. All toxins were shown to inhibit each of these enzymes but with varying degrees of potency (Figure 3). Consistent with the preliminary study, the order of sensitivity was PP2a > PP5 > PP1. Although PP2a was always 2- to 5-fold more sensitive than PP5, the relative order of potency to the toxins remained the same for each enzyme: nat. DTX1 > nat. OA > nat. DTX2 ≈ syn. DTX2 > syn. 2-*epi*-DTX2. Respective IC_50_ values (nM) for PP2a were 0.31, 0.47, 0.99, 1.35, and 137, and for PP5 were 1.3, 2.3, 5.2, 4.0, and 541 (Table 2). For PP2a, TEFs (relative to OA) were 1.5 (nat. DTX1), 0.34–0.47 (nat. and syn. DTX2), and 0.003 (2-*epi*-DTX2). For PP5, TEFs (relative to OA) were 1.8 (nat. DTX1), 0.44–0.58 (nat. and syn. DTX2), and 0.004 (2-*epi*-DTX2).

The sensitivity of PP1 was much less (1 to 2 orders of magnitude) with a different order of potency for the DSP toxins: nat. OA > nat. DTX1 > nat. DTX2 ≈ syn. DTX2 >> syn. 2-*epi*-DTX. Most notably, PP1 was less sensitive to nat. DTX1 relative to nat. OA. Respective IC_50_ values (nM) for PP1 were 25.2, 34.8, 76.4, 82.6, and 3110 (Table 2). For PP1, TEFs were 0.72 (DTX1), 0.33–0.31 (nat. and syn. DTX2) and 0.008 (2-*epi*-DTX2) (Table 2).

## 3. Discussion

Okadaic acid (OA) and dinophysistoxins (DTX) are algal toxins that can accumulate in seafood and cause diarrheic shellfish poisoning. In many countries, regulatory agencies monitor for these toxins and have established toxin-level thresholds that help ensure seafood consumer safety. For example, the European Union has set a limit of 160 μg/kg OA equivalents in shellfish meat, below which the product is deemed safe for human consumption. Since the potencies of each analog within a toxin class may vary, equivalents, or toxic equivalence factors (TEFs), are used to account for these differences. For the OA/DTX toxin class, TEFs have been determined via lethal doses in mice. According to expert opinion from the European Food Safety Authority (EFSA), derived TEFs for the OA/DTX class are: OA = 1, DTX1 = 1, DTX2 = 0.6. For DTX3 the TEF values are equal to those of the corresponding unesterified toxins (OA, DTX1, and DTX2) [22].

It is well established that OA is a protein phosphatase 2a and 1 inhibitor [8,18] with molecular modeling supporting this activity [11,15]. It has also been shown that OA inhibits PP5 [23,24] but much less is known about this interaction. Similarly, less is known about the interactions of DTX1 and DTX2 with PPs. Analogs of OA, DTX1, and DTX2 vary in C31 and C35 methylation and stereochemistries [1,25]. Two important molecular recognition sites have been identified; a bimetallic groove that accommodates the carboxylate-bearing C3–C12 spiroketal head region of OA/DTX and a distal hydrophobic groove that hosts the terminal C30–C38 tail spiroketal [15,16]. The carboxylic acid head region of OA that binds close to the active site (Tyr265 and Arg89 in PP2a and Tyr272, Arg96, and His125 in PP1) [11,17] appears to be the primary pharmacophore as structural modifications at C1 and C2 can reduce inhibitory potencies by several orders of magnitude [14,18]. On the other hand, structural and stereochemical modifications within the tail region of OA/DTX have a lesser effect on binding to the hydrophobic groove, with PP2a contact points at His191 and Gln122 [17]. Minor modifications in the tail region provide a structural basis for the reduced toxicity of DTX2 [17] and may be useful targets for distinguishing PP selectivity. 

The current study confirms that only three PPs (PP2a, PP1, and PP5) are sensitive to OA/DTX. On the other hand, PTPs and dual specificity phosphatases (i.e., DUSP22, TMDP, VHR) are not sensitive. PP5 is abundant in the nucleus and cytoplasm of eukaryotes [23], and contains a tetratricopeptide repeat (TPR) [13]. It has high catalytic domain sequence homology to PP1 and PP2a (35%–45%) [23], and functions to regulate the cell cycle and glucocorticoid receptor activity [26,27]. The Protein Data Bank has several listings for the catalytic domain of PP5 [13,28] that will be helpful for modeling efforts that have yet to be undertaken. Furthermore, potent inhibition of PP5 by the OA/DTX toxin in addition to the more widely known PP2a and PP1 may have confounded previous interpretations of cell signaling experiments and may contribute to other mechanisms of toxicity and adverse effects by the OA/DTX toxins [29]. 

The IC_50_ values for OA on PP2a, PP5, and PP1 are within the range of other published reports [8,17,18,19,23,24] as is the IC_50_ for DTX2 on PP2a (2.81 ng/mL or 3.5 nM [19]). PP2a was the most sensitive to the OA/DTX toxins, followed by PP5 and then PP1. PP2a was 2- to 5-fold more sensitive than PP5 and 10- to 100-fold more sensitive than PP1. For PP2a and PP5, the order of potency of the natural analogues was: DTX1 > OA > DTX2. On the contrary, for PP1 the order of potency was OA > DTX1 > DTX2, suggesting that methylation at C35 in the tail region of the molecule differentially affects PP1 versus PP2a and PP5. 

Although these data have shown that OA is more potent towards PPs than DTX2, this does not appear to be the case for cytotoxicity potential, where OA, DTX1, and DTX2 were all of equal cytotoxic potency. Tentatively, these differences may be explained by differential uptake into the cells, differential metabolism, and/or the heterogeneous protein phosphatase profile within this particular cell type. 

Detailed experiments quantifying the inhibitory effects of natural OA/DTX toxins may be helpful to regulatory agencies when implementing toxic equivalence factors (TEFs). In the current study, the TEF for DTX2 based on the three PPs ranged between 0.33 and 0.47 (relative to OA), which are similar to the 0.6 TEF proposed by Aune et al. [19] and the 0.48 TEF determined by Huhn et al. [17] using data obtained from Aune et al. [19] and Takai et al. [18]. The TEF used by the European Food Safety Authority (0.6) is based on LD_50_ values from exposed mice [19,22]. In contrast, the TEF for DTX2 using the cytotoxicity method was 1.58, poorly correlating with all available in vitro PP and in vivo studies. For DTX1, the TEF values appeared to be dependent on the PP sub-type. For PP2a and PP5, DTX1 is of greater potency than OA (TEFs 1.5 and 1.8; respectively), which is consistent with the TEF value of 1.58 proposed by Huhn et al. [17]. However, for PP1, which is in general orders of magnitude less sensitive to the OA/DTX toxins, the TEF value was 0.72. The TEF for DTX1 currently used by the European Food Safety Authority is 1.0 (equal potency to OA). Similar to DTX2, TEF determinations for DTX1 using the cytotoxicity model are also unreliable (TEF 0.87). 

The use of synthetic 2-*epi*-DTX2 (a C2 stereoisomer of DTX2) [30,31,32,33,34] elegantly tested the importance of the terminal spiroketal domain in binding to the Tyr265 and Arg89 of PP2a and Tyr272, Arg96, and His125 in PP1 [11,17]. Relative to DTX2, 2-*epi*-DTX2 potency was substantially reduced (1–2 orders of magnitude) towards PP enzymes and T lymphocytes. This C2-dependent shift in potency is consistent with the removal of the C2 hydroxyl group of OA that resulted in a 30-fold reduction in potency towards PP2a and a 6-fold reduction towards PP1 [18]. These data further corroborate modeling studies that suggest the C1–C13 head region of the molecule is the primary pharmacophore for PPs [14,18] and that PP2a is differentially sensitive to C2 molecular modification for both OA and DTX2. On the other hand, epimerization at C19 of OA has been shown to have no effect towards PP2a but reduced inhibitory potency towards PP1 [35], suggesting that future studies could design highly specific PP inhibitors.

In conclusion, this study has shown that there are three known PPs (PP2a, PP5, and PP1) that are differentially sensitive to OA/DTXs, but that these toxins do not affect the activity of a variety of PTPs and dual-specificity phosphatases. Natural analogs of OA, DTX1, and DTX2 demonstrate that changes in methylation of the tail C31 and C35 region weakly affect potency whereas molecular modifications to the head region (i.e., C2 epimerization) affect potency by more than two orders of magnitude. This study is also the first to provide detailed information on the effects of the OA/DTX toxin class towards PP5. Collectively, these studies have helped elucidate the interactions between OA/DTXs and their various phosphatase targets, which is helpful for researchers using these compounds as pharmacological tools and for coastal resource managers that are responsible for regulating toxin levels in seafood intended for human consumption. Furthermore, these data may serve as the framework for future molecular modeling studies and/or the development of highly specific phosphatase inhibitors.

## 4. Materials and Methods

### 4.1. Toxins

Synthetic DTX2 (ca. 95% purity, 0.42 mg) and synthetic 2-*epi*-DTX2 (0.83 mg) were produced as described elsewhere [21,30]. Natural (nat.) DTX1 (80% purity, 0.5 mg) was isolated from a *Dinophysis* bloom [25]. Synthetic DTX2, 2-*epi*-DTX2, and nat. DTX1 were dissolved to 2.5 mM in DMSO. Naturally-produced DTX2 and OA (in methanol) were obtained as certified reference materials from NRC Canada, Halifax, NS, Canada, dried down under N_2_ gas, and dissolved in DMSO (to final concentrations of 50 μM and 17 μM, respectively). All toxin dilutions were performed in DMSO. All toxin exposures were ≤1% vehicle (*v*/*v*).

### 4.2. Cytotoxicty Assays

To determine the effect of OA/DTXs on cellular toxicity, Jurkat T lymphocyte cells were continuously exposed to toxins for a given time period and their viability determined [36]. The non-adherent human Jurkat T lymphocyte cell line (American Type Culture Collection *cat.* # TIB-152, Manassas, VA, USA) was grown in RPMI medium supplemented with 10% (*v*/*v*) fetal bovine serum (FBS). Cells were maintained in humidified 5% CO_2_ in air at 37 °C and subcultured with fresh medium at an inoculum ratio of 1:10 every 5 to 7 days by transferring 1 mL of cells to 9 mL of fresh supplemented medium in 75 cm^2^ screw cap culture flasks. Cells were seeded in a volume of 100 μL of the supplemented medium at a density of 30,000 cells per well in black, sterile, 96-well culture plates for 12–18 h to allow for recovery and settling. A range of final OA/DTX concentrations was then added for 24, 48, or 72 h of continuous exposure prior to assessment of cytotoxicity. Parallel vehicle controls comprising equivalent amounts of DMSO were used to normalize viability data for each treatment. Cellular viability/cytotoxicity was assessed using the MTS assay (Promega Biosciences, San Luis Obispo, CA, USA; *cat.* no. G5421). Like other tetrazolium-based assays, 3-(4,5-dimethylthiazol-2-yl)-5-(3-carboxymethoxyphenyl)-2-(4-sulfophenyl)-2*H*-tetrazolium (MTS) in the presence of an electron coupling reagent (phenazine methosulfate; PMS) measures cellular viability by determining the activity of mitochondrial dehydrogenases [37]. Following exposure of the cells to OA/DTX for a specified period of time, each well received 10 μL of a PMS/MTS (1:20) solution. Cells were incubated for 3–4 h, after which absorbance readings at 485 nm were obtained using a FluoStar microplate reader (BMG Lab Technologies, Ortenberg, Germany). Data are presented as means ± SE of at least three separate experiments. In addition, each cytotoxicity experiment was performed using duplicate wells. Cytotoxicity data were blank-corrected and normalized to the control (% viability). EC_50_ and 95% confidence intervals were calculated using three-parameter, variable slope, non-linear regression analysis (GraphPad Prism, ver. 5.0c, San Diego, CA, USA).

### 4.3. Protein Phosphatase Inhibition Assays

Preliminary studies were conducted by Millipore (EMD Millipore Corporation, Darmstadt, Germany) using their PhosphataseProfiler that assessed the activities of 21 human and yeast phosphatases in the presence of DTX2. Phosphatase sensitivities to DTX2 were more extensively characterized using 96-well fluorescence-based assays in the presence of various natural and synthetic OA/DTXs [38,39]. Briefly, the assay tests the ability of toxins to inhibit the activity of purified PP2A (EMD Millipore Corporation, Darmstadt, Germany, *cat.* #14-111; human red blood cells), purified PP1 (EMD Millipore Corporation, Darmstadt, Germany, *cat.* #14-110; rabbit skeletal muscle), or purified PP5 (EMD Millipore Corporation, Darmstadt, Germany, *cat.* #14-778; recombinant enzyme expressed in *E. coli* cells) against the fluorimetric substrate, 6,8-difluro-4-methyl umbelliferyl phosphate (DiFMUP; 50 μM final) (Invitrogen ThermoFisher Scientific, Waltham, MA, USA, *cat.* #D6567). DiFMUP solutions were preincubated with toxins or vehicle controls (all containing 1% DMSO final) for 10 min prior to addition of one of the PP enzymes (0.4, 0.4, and 0.02 units/well for PP2a, PP1, and PP5). PP2a assays were performed in Tris-HCl (40 mM, pH 8.4), MgCl_2_ (34 mM), EDTA (4 mM), and DTT (4 mM) [18], PP1 assays were performed in Tris-HCl (100 mM, pH 7.5), MnCl_2_ (0.5 mM), EDTA (0.2 mM), DTT (4 mM) and BSA (0.4 mg/mL) [40], and PP5 assays were performed in HEPES (57 mM, pH 7.2), NaCl (150 mM), EDTA (1 mM), MnCl_2_ (1 mM), DTT (0.167 mM), glycerol (0.83%), BSA (0.167 mg/mL), Brij-35 (0.002%) (EMD Millipore Corporation, Darmstadt, Germany, pers. comm.). All reactions (50 μL) were performed in black, 96-well, half area plates (Corning Incorporated, Corning, NY, USA) for 2 h at 35 °C with 5 s orbital shaking (ca. 600 rpm) just prior to each reading (every 2 min). PP activity was determined by measuring the fluorescence (excitation 360 nm, emission 470 nm) of each well using a fluorometric plate reader (Fluostar, BMG Lab Technologies, Ortenberg, Germany). All data (mean ± SE, *n* ≥ 3) are presented as normalized percentages relative to vehicle controls (100%). IC_50_ determinations were calculated using four-parameter, variable slope, non-linear regression analysis (GraphPad Prism, ver. 5.0c, San Diego, CA, USA).

## Figures and Tables

**Table 1:** Calculated EC_50_ values and relative potencies for natural OA, DTX1, and DTX2, and synthetic DTX2 and 2-*epi*-DTX2, based on T lymphocyte cytotoxicity.

Toxin	24 h			48 h			72 h			Rel. Potency (as per 48 h Data)
	EC_50_ (nM)	95% Confidence Intervals	*n*	EC_50_ (nM)	95% Confidence Intervals	*n*	EC_50_ (nM)	95% Confidence Intervals	*n*	
**nat. OA**	32.8	19.4–55.3	3	19.5	11.7–32.4	3	9.16	5.43–15.5	3	1.000
**nat. DTX1**	24.6	8.60–70.2	2	22.2	11.6–42.5	2	11.4	7.39–17.6	2	0.87
**nat. DTX2**	22.8	11.1–46.6	3	12.3	6.71–22.5	3	5.52	3.55–8.61	3	1.58
**syn. DTX2**	21.8	11.7–40.7	3	18.2	10.8–30.6	3	15.4	10.2–23.0	3	1.07
**syn. 2-*epi*-DTX2**	4750	2010–11,200	3	4260	1710–10,600	3	3740	1880–7470	3	0.004

**Table 2:** Calculated IC_50_ values and relative potencies for natural OA, DTX1, and DTX2, and synthetic DTX2, and 2*-epi*-DTX2, based on PP2a, PP1, and PP5 inhibition.

Toxin	PP2a				PP1				PP5			
	IC_50_ (nM)	95% Confidence Intervals	*n*	Rel. Potency	IC_50_ (nM)	95% Confidence Intervals	*n*	Rel. Potency	IC_50_ (nM)	95% Confidence Intervals	*n*	Rel. Potency
**nat. OA**	0.466	0.402–0.539	3	1.000	25.2	18.2–34.8	3	1.000	2.30	1.66–3.17	3	1.000
**nat. DTX1**	0.306	0.283–0.330	4	1.523	34.8	21.9–55.2	4	0.724	1.30	1.06–1.66	4	1.769
**nat. DTX2**	0.987	0.844–1.16	3	0.472	76.4	57.9–101	3	0.330	5.25	3.96–6.98	4	0.438
**syn. DTX2**	1.35	1.12–1.63	3	0.345	82.6	55.4–123	3	0.305	3.95	3.18–5.00	4	0.582
**syn. 2-*epi*-DTX2**	137	106–178	3	0.003	3110	2080–4660	3	0.008	541	433–675	4	0.004

## Supplementary Materials

The following are available online at www.mdpi.com/1660-3397/14/11/207/s1, Figure S1: Effect of natural DTX-2 on phosphatase activity.

## Abbreviations

DTX1	Dinophysistoxin-1
DTX2	Dinophysistoxin-2
EC_50_	50% Maximal Effective Concentration
IC_50_	50% Maximal Inhibitory Concentration
MTS	3-(4,5-Dimethylthiazol-2-yl)-5-(3-carboxymethoxyphenyl)-2-(4-sulfophenyl)-2H-tetrazolium
OA	Okadaic Acid
PBS	Phosphate Buffered Saline
SAR	Structure–Activity Relationship
TEF	Toxic Equivalence Factor
