# Supplementary material for: Structure–Activity Relationship Studies Using Natural and Synthetic Okadaic Acid/Dinophysistoxin Toxins
Source: Mar Drugs. 2016 Nov 4;14(11):207. doi: 10.3390/md14110207 (PMC5128750; doi:10.3390/md14110207)
Supplement: Supplementary file 1 [file marinedrugs-14-00207-s001.pdf]

Supplementary Materials: Structure-Activity Relationship Studies Using Natural and Synthetic Okadaic Acid/Dinophysistoxin Toxins

Michael J. Twiner, Gregory J. Doucette, Yucheng Pang, Chao Fang, Craig J. Forsyth and Christopher O. Miles

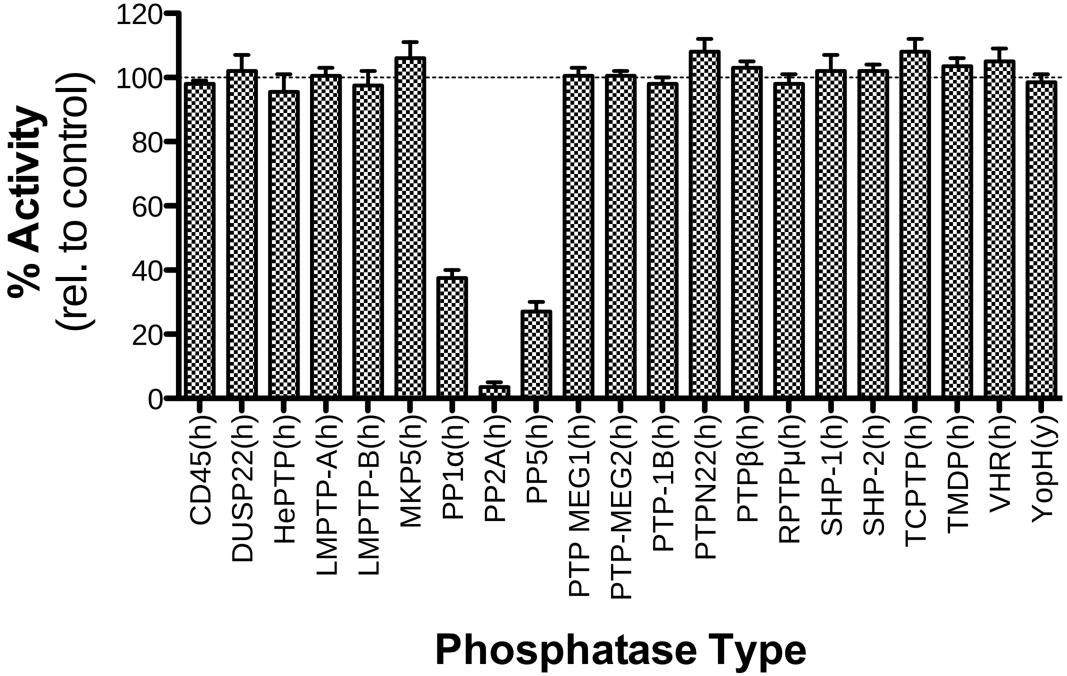

Figure S1. Effect of natural DTX-2 on phosphatase activity. As a part of the PhosphataseProfiler™ performed by EMD Millipore Corporation (Darmstadt, Germany), the activities of 21 different protein phosphatases, dual-specificity phosphatases and tyrosine phosphatases were tested in the presence of natural DTX2 (200 nM). All data (mean ± range; *n* = 2) were normalized to the control and illustrated as percent activity.
